# Investigating Maternal Perspectives of Breastfeeding Support Targeted Towards Fathers in the Milk Man Mobile App Intervention

**DOI:** 10.1007/s10995-023-03616-5

**Published:** 2023-03-15

**Authors:** Becky K White, Roslyn C Giglia, Sharyn K Burns, Jane A Scott

**Affiliations:** 1 School of Population Health, Curtin University, GPO Box U1987, Perth, 6845 Australia; 2 Foodbank WA, Perth, 6105 Australia; 3 Collaboration for Evidence, Research and Impact in Public Health (CERIPH), Perth, Australia

**Keywords:** Breastfeeding, Father, Mother, Mobile health

## Abstract

**Background:**

The support of her infant’s father is one of the most important factors influencing a mother’s breastfeeding success, and an increasing number of interventions are targeted towards fathers. Engaging fathers as agents to influence a maternal behavior is potentially problematic, yet few studies report on maternal experiences.

**Objective:**

This study aims to explore mothers’ perspectives of their partners’ use of Milk Man, a father-focused breastfeeding smartphone app, and the acceptability of this approach.

**Materials and methods:**

New mothers (N = 459) whose partners had access to the app completed a questionnaire at six weeks postpartum. These data were used to determine knowledge, use and perspectives of the app. A sentiment analysis was conducted on responses to an open-ended question seeking maternal perspectives of the app.

**Results:**

Just over a quarter of mothers (28%) had been shown something from the app, and 37% had discussed something from Milk Man with their partner. There were 162 open-ended responses related to mothers’ perspectives of the app. Relevant responses (*n* = 129) were coded to an overall sentiment node and then to a total of 23 child nodes (sub-nodes). Most comments were positive (94), with a smaller number either negative (25) or neutral (21). Negative comments related to the usability of the app and not its intent or content.

**Conclusion:**

Mothers found the father-focussed breastfeeding app to be acceptable. When designing interventions targeting one group to affect the behaviour of another, inclusion of measures to gain the perspectives of both should be seen as an imperative.

**Supplementary Information:**

The online version contains supplementary material available at 10.1007/s10995-023-03616-5.

## Significance

*What is already known on this subject?*

Breastfeeding has significant health benefits for infants and mothers. Fathers are important supports for breastfeeding mothers and influence both the decision to breastfeed, and breastfeeding duration. Many father-focussed breastfeeding interventions have shown positive breastfeeding outcomes, but little is known about maternal perspectives of these interventions.

*What this study adds?*

This study adds to the literature by seeking maternal perspectives on a father-focussed breastfeeding intervention. It demonstrates that mothers found the intervention acceptable, and highlights the importance of including the perspectives of mothers on interventions designed to influence their behaviour, but targeted at their male partner.

## Background

Despite well evidenced and compelling benefits for both infants and mothers (Chowdhury et al., 2015; Victora et al., 2016), less than 25% of Australian infants are exclusively breastfed to the recommended six months of age (Australian Bureau of Statistics, 2017a). While many factors affect a woman’s decision to initiate breastfeeding and how long she breastfeeds for, an important underlying predictor of breastfeeding success is self-efficacy, or the belief a person holds that they are able to complete a task and that it will lead to a desired outcome (Luszczynska & Schwarzer, 2005). Maternal breastfeeding self-efficacy is an important predictor of breastfeeding initiation, exclusivity and duration (Tuthill et al., 2015) (Blyth et al., 2002). Mothers who have a high level of confidence in their ability to breastfeed, hence a high level of breastfeeding self-efficacy, are more likely to breastfeed (Blyth et al., 2002) and to breastfeed for longer (Lau et al., 2018). Research shows that the support of a woman’s partner is a strong predictor of breastfeeding self-efficacy (Hinic, 2016; Li et al., 2022) and crucial to breastfeeding success. This support can take a number of forms and may be practical, physical and/or emotional (Rempel et al., 2017).

In Australia, there is compelling empirical evidence, reinforced over time, of the importance of fathers in supporting breastfeeding. Women who perceive that their partner is supportive of breastfeeding are more likely to initiate breastfeeding, to exclusively breastfeed, and to breastfeed for longer than those who perceive their partner to prefer bottle feeding or to be ambivalent about how they feed their baby (Ayton et al., 2015; Scott, Binns, Oddy, & Graham, 2006). Similarly, international research highlights that paternal positive attitude, involvement and support increase breastfeeding duration (Mahesh et al., 2018; Wang, Guendelman, Harley, & Eskenazi, 2018).

Informed by this evidence, an increasing number of breastfeeding interventions have sought to influence maternal breastfeeding outcomes by targeting the father with information and support (Abbass-Dick et al., 2017; Bich, Hoa, & Målqvist, 2014; Mitchell-Box & Braun, 2012; Su & Ouyang, 2016). Yet the action of upskilling fathers to influence a maternal behavior is one that could potentially cause conflict between a couple. While this is an under-researched area, mothers have shared, via popular media, pressures they have experienced when their partner’s breastfeeding views conflict with their own (Gagnon, 2017, June 1st), as well as the way pressure from wider society can negatively affect maternal confidence (Montgomery, 2018, September 11th). Care must be taken to develop interventions that reinforce the autonomy of the mother, while empowering the father in his role as the supporter. Breastfeeding interventions should inform and educate fathers of the importance and benefits of breastfeeding so they can support and encourage their partner (Abbass-Dick et al., 2019). Yet, if this support crossed into persuasion, or direction, the intervention potentially could not only have a negative effect on breastfeeding, but could also introduce conflict to the couple’s relationship (Johnson & Slauson-Blevins, 2022). Despite the complexity in designing interventions which are targeting one group to have an effect on the behavior of another, to our knowledge no father-focused breastfeeding intervention study has reported the maternal perspectives of the intervention.

## Objectives

The aim of this research is to explore mothers’ perspectives of their partners’ use of Milk Man, a father-focused breastfeeding smartphone app, and the acceptability of the approach. The app aimed to engage fathers with information and conversation about breastfeeding, and develop breastfeeding self-efficacy in both parents by facilitating shared conversations and problem solving.

## Methods

Milk Man was developed for use in the Parent Infant Feeding Initiative (PIFI), a factorial randomized controlled trial (RCT) that evaluated the effectiveness of two father-focused breastfeeding interventions on breastfeeding duration, both singly and in combination (Scott et al., 2021). The project was approved by the Curtin University Human Research Ethics Committee (HR 82/2014; 14 May 2014). One intervention provided access to the Milk Man app and the second intervention included a single father-focussed antenatal breastfeeding class facilitated by a volunteer peer facilitator (Reference removed for blinding).

This study relates to quantitative and qualitative data collected from mothers involved in an intervention group which provided access to the Milk Man app. The app contained a comprehensive information library, a conversation forum and used push notifications and gamification as motivators for app use (White et al., 2016a). App content was carefully developed to empower fathers as breastfeeding supporters, without detracting from maternal autonomy and empowerment associated with breastfeeding. A key focus included encouraging fathers to ask their partners what they thought about different breastfeeding-related issues and how they could best support them. General information about breastfeeding, similar to that found on health authorities’ websites, was included and couples were encouraged to talk to a health professional if they were experiencing difficulty.

### Participants and Setting

For the broader RCT, 1426 heterosexual couples were recruited from hospital-based antenatal classes in Perth, Western Australia between August 2015 and December 2016; 730 couples were randomized to an intervention group that had access to Milk Man. All couples gave informed consent to participate. Most couples were expecting their first child, as in Australia few couples attend antenatal classes for the birth of subsequent children. While the interventions were targeted at the father, participants were recruited as a couple and were eligible to participate in the study if the father owned a compatible smartphone (iOS or Android); lived in Western Australia; had internet access; had English literacy; and if both parents intended to co-parent their child.

Survey data for the RCT were collected via questionnaires from both parents at recruitment, and 6 and 26 weeks postpartum. The baseline questionnaire was provided in a paper format and subsequent questionnaires were delivered and completed online using Qualtrics software (Qualtrics, Provo, UT). Different questionnaires were completed by fathers and mothers and included closed and open-ended questions specific to their experiences.

Of the 730 fathers randomized to receive Milk Man, 586 (80%) downloaded the app. Participants in this study are 459 mothers whose partners had access to Milk Man from approximately 32 weeks gestation until six months postpartum and who responded to the six-week follow-up questionnaire. Demographic information was collected in a separate questionnaire, at baseline, and this information was available for 416 (90.6%) of these mother. Not all women provided answers to all of the process evaluation questions and reasons for mothers choosing not to complete the survey are unknown. All responses given are included in this analysis.

### Measures

In addition to a variety of questions and scales related to breastfeeding and parenting practices (Reference removed for blinding), the six week questionnaire included questions specific to the app use and content, informed by the Mobile App Rating Scale (Stoyanov et al., 2016) and guided by the evaluation framework developed for the Milk Man app (White et al., 2016b).This paper reports on quantitative and qualitative data collected as part of the mothers’ six week questionnaire, with the qualitative component being the core focus of this paper. Open-ended app-related questions (n = 6) were asked at six-weeks postpartum for mothers whose partners had access to Milk Man (supplementary file 1). These focused on the usefulness of the app, mothers thoughts about the app, and any discussions they had with their partner about the app.

Closed-ended questions, which included Likert scale and multiple-choice responses, were asked to determine if participants had been aware of their partner using the app; if they had been shown anything in the app or had any conversations about the app; and if they had used the app themselves. If they responded positively to being shown something, or to having a discussion about something in the app, an open text response option was provided to allow them to record what it was. Mothers were also asked an open-ended question exploring their overall thoughts about the app.

### Data Analysis

As preliminary data analysis exploring fathers use of the app revealed paternal app usage was concentrated in the weeks around the time of birth (White et al., 2018), this study reports on data collected at six weeks postpartum. Responses to quantitative questions are presented as percentages and frequencies, with the denominator representing the number of participants answering each individual question.

Maternal perspectives about the appropriateness of targeting fathers with breastfeeding information via the Milk Man app were explored by a sentiment analysis of the final open-ended question. Sentiment analysis involves allocating an overall sentiment node (of positive, negative or neutral) to qualitative data (Mantyla et al., 2018). It can be useful when seeking an overall understanding of sentiment with a large amount of qualitative data. A pragmatic paradigm was adopted, allowing the qualitative data to provide a richer interpretation of the quantitative findings (Liamputtong, 2013).

The sentiment analysis explored responses to the open text questions to provide insights into mothers’ overall thoughts about Milk Man. The analysis involved initially coding a participant’s comments to a top-level sentiment node of positive, negative or neutral depending on the overall nature of the response. In total, 162 comments were recorded, with 33 comments coded as not applicable (N/A) (including the following responses: N/A; I haven’t used it; no comment; and I’m not sure). These comments were excluded from the remainder of the analysis, leaving 129 comments for analysis. Comments were then inductively coded to relevant sub-nodes, or child nodes (for example – Sentiment: Positive, Child nodes: good for dads; helpful / informative). The data were initially coded by one researcher and then reviewed by another to ensure confirmability (Bryman, 2004). Both researchers had expertise in qualitative data analysis. Consensus of coding to the nodes among the two reviewing authors was reached through discussion and review. The NVivo Qualitative Data Analysis software package 12 (QSR International) was used to manage the data. All comments are reported as they were written by participants.

## Results

Of the participants for whom demographic data were available (n = 416/459; 90.6%) most were born in Australia or New Zealand, were university educatedand aged between 30 and 34 years (Table 1).

**Table 1:** Characteristics of participant mothers (*n* = 416)

Characteristics	*n* (%)
Age in years	
< 30	108 (26)
30–34	205 (50)
≥ 35	101 (24)
**Education**	
High school or trade	107 (26)
Undergraduate university education or higher	309 (74)
**Country of birth**	
Australia or New Zealand	280 (67)
United Kingdom or Eire	49 (12.5)
Africa of Middle East	12 (3)
Asia	43 (10)
Other	31 (7.5)

Note: Other category was for participants whose country of birth was outside of the nominated regions

Missing values are: Age = 2; Country of birth = 1

Over half of mothers (59%, *n* = 245/418) were aware of their partner looking at or using the app but 92% *(n* = 384/418) of mothers had not used the app themselves. A total of 28% (*n* = 116/418) of mothers said their partner had shown them something from the app, with 101 open text answers provided as to what they were shown. The most common feature was the discussion forum (*n* = 47). This was followed by the information library (in general) (*n* = 19), different aspects of the app design and functionality (for example: how it looked, or how it worked) (*n* = 16) and library information specific to breastfeeding (*n* = 12).

Just over one third of participants (37%, *n* = 155/416) indicated they had discussed something from the app with their partner. The main discussion topics related to breastfeeding in general (*n* = 37), and to the conversation forum (*n* = 45). This included comments about the different content in the conversation, fathers’ experience of the forum and the effect of the forum on the father. Most participants reported their partners finding the app valuable, however barriers to the app were also discussed. Most mothers indicated their partner found the app useful as they were able to discuss salient issues with other new fathers. For example, some reported benefits of the app included: “*He found it helpful to hear what other guys are struggling with*.” “*He also told me about some of the other men’s experiences with their partners and we learnt from their advice etc.*”

Mothers also suggested the app generated discussion around a range of topics including mastitis, alcohol and breastfeeding, breastfeeding techniques and support. The app facilitated discussion between many parents about planning for breastfeeding. “*How long we will try and breastfeed for. Advantages of breastfeeding*.” “*What he can do to help when I am feeding.*” “*The importance of breastfeeding for baby health*.”

However, while many participants reported their partners using the forum in positive ways, two participants reported that the conversation forum had less activity than their partner wanted and that this had negatively affected his experience. “*He thought there would be more interaction between the dads. Was a little disappointed that there wasn’t more of a chat feature on it*.”

A smaller subset of participants provided comments that were included in the sentiment analysis. There was no difference in the sociodemographic characteristics of women who commented on Milk Man compared to those who didn’t comment with the exception of education. In which case, university educated women were more likely to have commented than those who had not received a university education. Table 2 shows demographic characteristics of the subset of participants who provided comments included in the sentiment analysis.

**Table 2:** Characteristics of mothers making RELEVANT comments related to Milk Man

	No Comment		Commented		
Characteristic	N	%	N	%	P value ^a^
Mother’s age (years)					0.544
< 30	111	28.4	37	24.5	
30–34	185	47.3	79	52.3	
≥ 35	95	24.3	35	23.2	
Mother’s education					**0.007**
High school/vocational	115	27.3	19	15.4	
Some or completed university	306	72.7	104	84.6	
Mother’s country of birth					0.282
Australia/ New Zealand	271	64.4	88	71.5	
United Kingdom/ Ireland	50	11.9	15	12.2	
Asia	56	13.3	9	7.3	
Other	44	10.5	11	8.9	
IRSAD ^b^ quintile					0.876
1 – most disadvantaged	9	2.0	4	3.4	
2	15	3.4	4	3.4	
3	93	21.1	24	20.2	
4	113	25.7	27	22.7	
5 – least disadvantaged	210	47.7	60	50.5	

^a^ Chi-square

^b^ IRSAD: Index of Relative Social Advantage and Disadvantage

The 129 comments included in the sentiment analysis generated a total of 23 child nodes with 354 individual references. Most comments were coded overall to a single top-level node and then subsequently coded to one or more child nodes. The number of comments under each sentiment, as well as each child node category, is shown in Table 3.

**Table 3:** Categories derived from responses to question asking mothers what they thought about the Milk Man app (*n* = 129)

Sentiment node	Child nodes (categories)	*n* (%)
Positive		94 (73%)
	Helpful / informative	48
	Good for dads	43
	Makes dads feel more involved	22
	Good support for dads	21
	Mums feel more supported	13
	General	10
	Entertaining / gamification	7
	Requests for app to be publicly available	2
Negative		25 (19%)
	Not useful	7
	Hard to use	6
	Not enough activity in conversation	4
	Too basic	3
	Prefer real-life	3
	Overwhelmed by technology	2
	Gamification	1
	General	1
Neutral		21 (16%)
	General	10
	Couldn’t access app	5
	Not enough time	5
	Lack of internet access	1

Note: The general category included comments that didn’t specifically have any direct relevance to any other categories. For example, a comment coded as general in the neutral section read ‘I haven’t used it enough to be able to give a useful opinion’

Most of the comments about the app were coded as a positive sentiment (*n* = 94, 73%). The top two categories were comments stating the app was ‘good for dads’ and that it was ‘helpful or informative’. There were no negative comments from mothers about the intent of the app or the appropriateness of targeting fathers. Table 4 contains examples of comments coded to each sentiment, and the child node codes assigned to each comment.

**Table 4:** Examples of sentiments and child node categories

Sentiment	Examples	Codes assigned
Positive	*I think it is an excellent tool to support Men/fathers in participating in the breastfeeding experience and feeling part of providing for baby. It provides them information in an easy way which makes it more likely to be digested and called upon. A fantastic initiative - great work! Having the format be an app is excellent for engagement for modern men.*	Helpful / informative. Good for dads. Make dads feel more involved.
	*I do not know how often he uses it, but there have been the occasional times that I’m worried about something and he encourages me with something he’s read on the app.*	Helpful / informative. Good for dads. Mums feel more supported.
	*It is an app that has allowed my husband to be both informed and confident to support me with breastfeeding. He was so helpful in the hospital in establishing breastfeeding and attachment - reminding me of techniques which I believe allowed me to relax more and be successful in establishing exclusive breastfeeding with our baby.*	Helpful / informative. Good for dads. Mums feel more supported.
Negative	*The knowledge base is not large, so doesn’t allow for a lot of information to search for. My husband prefers to be able to search for information, preferably academic articles.*	Not useful.
	*I (assumed?) it was just for the dads so haven’t look at it myself. I know my husband has said he hasn’t found it useful, he is not really an app/forum/game kind of person mind you, he would much rather meet and chat to other dads over a beer or something like that.*	Not useful. Prefer real life.
	*My husband feels it is too complicated so we have not used it as a resource as much as I feel we could have*	Hard to use.
Neutral	*My husband hasn’t used it as he couldn’t find the code to log into it with!*	Couldn’t access app.
	*Don’t know. Partner says it was somewhat helpful. Recalling advice from hospital Midwife & CHN [Child Health Nurse] was more helpful.*	General

The majority of participants indicated that their partner benefited from the app. Access to information provided via the app enabled fathers to prepare for the baby and also provide support after the birth. Participants reported fathers reading through the information library, visiting linked resources, and valuing the information targeted to them and the autonomy to be able to do their own research. “*I know my partner found it useful with the information before we had the baby. He spent quite a bit of time reading though the resources and I think it helped prepare him a little bit*.” “*My partner really liked it and got a lot out of it. It was good for him to have his own source of information separate from anything I had access too*.”

Some participants reported their partner was initially unsure about the value of the app, however, did find it useful and enjoyable once they tried it. “*My partner was cynical about it but actually found it useful and did enjoy using it and reading things on it*.”

One participant reported that while she initially thought the app was not a very good idea, she thought that being part of the research had impacted on the support her husband could give her and that she had benefited from this support.

*The app is a dumb idea it duplicates the information and services already available on the internet. BUT: The mere fact that Dads were a focus of this program I think helped my husband to realise Breastfeeding is no walk in the park, and he probably did a LOT more chores around the house and supporting me because of being prepped*.

A few participants also commented directly on the gamification strategy and while some considered this was important for engagement, especially the competitive element, it was also considered a barrier for some men. “*I like the idea of an app with information targeted at fathers, but I don’t think it’s necessary to make it a ‘game’ with points. I think it stopped my husband from utilising it as much as he could have*.” “*The one thing he did show me was everyone in his group he was able to compete with the others and get points and males being males, I think that’s a clever idea to get them involved.*”

Some participants commented that the app had raised new issues for their partner to consider including about breastfeeding, about sourcing support for themselves and how to support and encourage their partner. “*My partner has used it and it’s made him think about things he may otherwise not have thought about.”*

The 25 (19%) negatively coded comments were related to specific functions or

usability of the app; the child node with the most allocations was ‘not useful’ (*n* = 7) followed by ‘hard to use’ (n = 6). Some participants suggested the app was not useful for their partner as they didn’t experience issues with breastfeeding, or they were supported by lactation consultants. For example:

*May be useful for people with ongoing breastfeeding issues or spousal issues related to breastfeeding, but I haven’t experienced that so it hasn’t been useful for me*.

*My husband said we ended up getting all the information from Lactation Consultants, midwives and from my googling etc, I think having the first few weeks a total blur and trying to keep head above water, he didn’t think about the app much. Sorry!*

Only a few participants suggested their partner found the app hard to use: “*My husband feels it is too complicated so we have not used it as a resource as much as I feel we could have*.”

Overall, participants felt Milk Man was helpful and an appropriate strategy to target fathers during the perinatal period. The app was considered an important tool which provided good information and engaged fathers in breastfeeding decisions and support. “[Milk Man] *Gave him information so he could help work through/suggest/solve breastfeeding problems. Partner was involved with breastfeeding experience*.”

Importantly, some of the responses offered specific detail about how the app had helped their partner to support them as a mother, and with breastfeeding.

“*It was surprisingly helpful. That first night was such a struggle and our baby and I just didn’t understand how to latch. My husband opened the app with the info on how to do this and together he helped us figure it out. Whilst I know he would have been there to help me either way I don’t think he would have felt like he could truly help and be involved like he was or have known where to go for this information.”*

The above comment example demonstrates the value of the app in both empowering fathers with information provision and ways he can provide support, and how that support impacts on a mother’s experience.

## Discussion

Pregnancy and childbirth is a time of many new experiences for first-time parents and the Milk Man app was useful in facilitating conversations about aspects of parenting, in particular breastfeeding, which couples may not have considered previously. This is an important finding as parents that work together to prepare for challenges and changes in the perinatal period fare better in terms of mental health outcomes than those who do not (Colquhoun & Elkins, 2015). A key finding of this study is that no mother reported negatively on the intent of the app, with most mothers reporting the app helped to support their partner, and, in turn, helped their partner to support them.

Breastfeeding is a new skill to be learned by first-time mothers and many women experience difficulty in establishing breastfeeding. Their journey may be emotional and women have reported feelings of guilt and distress if things are not going well (Burns et al., 2010; Constantinou, Varela, & Buckby, 2021; Guyer, Millward, & Berger, 2012; Russell, Birtel, Smith, Hart, & Newman, 2021). Mothers may even feel they are failing or are not a good mother if breastfeeding is unsuccessful (Jackson et al., 2021; Palmér, Carlsson, Mollberg, & Nyström, 2012). Partner support is vital for breastfeeding mothers, yet care needs to be taken to ensure that it does not undermine the woman’s confidence in breastfeeding, and that support is targeted towards increasing her self-efficacy (Johnson & Slauson-Blevins, 2022). Milk Man aimed to do this by consistently encouraging fathers to talk with their partner, and to ask how they could best support them. Although this paper does not report on maternal self-efficacy, other studies have shown that interventions targeted to the father in the perinatal period can impact positively on maternal self-efficacy (Hadian Shirazi et al., 2022).

Interestingly, the proportion of participant mothers reporting that they had discussed something from the app with their partners (37%) was lower than that reported in the earlier process evaluation of the app by fathers (54%) who had access to Milk Man (White et al., 2018). This suggests participant fathers may have been initiating conversations that originated from the app more often than their partners were aware. Other studies have reported fathers feeling left out of breastfeeding education, or of not understanding their role and how they can help (Mitchell-Box & Braun, 2012; Tohotoa et al., 2009) hence the number of reported conversations instigated by Milk Man described in this study is encouraging.

Further evidence of couples discussing the app was provided in the overlap of reported experiences. The conversation forum was a central part of the Milk Man app, which participant fathers used to facilitate social support in a variety of ways White et al., 2018). The Milk Man intervention was part of a large RCT with participants recruited over an 18-month period. As participant fathers were grouped within the app by the expected month of their baby’s birth, some of the conversation groups were relatively small (group numbers ranged from 16 to 47) which had an impact on the level of engagement by some fathers in this component of the app. Participant mothers also expressed these experiences when talking about their partner’s use of the app. Scaling-up the Milk Man app will create larger conversation groups, which should result in more fathers engaging in the conversation forum.

Although the app was designed with input from fathers only, it was led by a team of nutritionists, midwives and health promotion professionals who have significant experience in the development, implementation and evaluation of breastfeeding interventions for both fathers and mothers. Involving both parents in the consultation phase in interventions similar to Milk Man presents an interesting paradox. Breastfeeding interventions targeting fathers need to be at the same time be relevant to the needs of the father but mindful of the mother, taking an informed and careful approach to optimise acceptability to both parents. While some participant fathers suggested they preferred to source information elsewhere, most found it a useful way to source information, and to connect with other fathers (White et al., 2018). One of the benefits of a digital intervention is the ability to scale quickly and cost-effectively (World Health Organization, 2011).

These findings have broad implications for future practice. This study has demonstrated that an appropriately designed breastfeeding app intervention for fathers can be acceptable to mothers. Further research should be undertaken to better understand the effect of the intervention on a range of other aspects of parenting including mental wellbeing and partner support. Findings from this study, combined with evidence of fathers’ engagement with the app reported previously (White et al., 2018), provide compelling evidence of the acceptability of this approach and impetus to continue research in this area.

## Limitations

Only one third of respondent mothers offered comments on their perspectives of the app that were included in the sentiment analysis. While the responses provided mixed results, it is possible people who were more engaged with the app or felt more strongly about it (either positively or negatively) were more motivated to share their views. In addition, social desirability bias may have affected the nature of the comments provided to researchers. Comparison with 2016 Census Data shows that the study population was similar in age and ethnicity to the general population of Perth women in this age group. However, they were more highly educated than the general population with only 36% of women aged 20–39 years living in the Perth metropolitan area having a university degree compared with 74% in this study (Australian Bureau of Statistics, 2017b). This may have introduced a bias as these mothers may have strong social networks and high social capital, thus limiting the generalizability of these findings. Future studies may benefit from different recruitment processes and include a more concentrated focus on recruiting from a more representative sample.

## Conclusion

This study is significant as it describes how evaluation of maternal perspectives of a father-focused breastfeeding intervention can be incorporated to strengthen intervention design. When designing evaluation for breastfeeding interventions directed at those other than the mother, it is important to seek and understand maternal perspectives. We have demonstrated in this research that targeting breastfeeding education and support to fathers through the Milk Man smartphone app is an approach that is acceptable to, and deemed appropriate by, respondent mothers.

## Electronic supplementary material

Supplementary table

## Data Availability

Data available from authors on reasonable request.
