# Supplementary material for: Investigating Maternal Perspectives of Breastfeeding Support Targeted Towards Fathers in the Milk Man Mobile App Intervention
Source: Matern Child Health J. 2023 Mar 15;27(5):954–64. doi: 10.1007/s10995-023-03616-5 (PMC10115714; doi:10.1007/s10995-023-03616-5)
Supplement: Supplementary file 1 — Supplementary table [file 10995_2023_3616_MOESM1_ESM.docx]

**Supp File 1: Milk Man app-related questions. The following questions are from the six-week questionnaire that were asked of mothers whose partners had access to the Milk Man app.**

**-------------------------------------------------------------------------------------------------------**

As part of this research project, your partner has access to the Milk Man app.

1. Have you been aware of him using or looking at the app? Have you been aware of your partner using or looking at the Milk man app?
   1. Yes
   2. No
2. Have you used the app yourself?
   1. Yes, often
   2. Yes, occasionally
   3. Yes, a little
   4. No, not at all
3. Has your partner showed you anything from the app?
   1. Yes (go to 3.a.i)
      1. What information has he shown you? (open text)
   2. No (go to 4)
4. Have you had any discussions with your partner about anything from the app?
   1. Yes (go to 4.a.i)
      1. What have you discussed? (open text)
   2. No (go to 5)
5. How helpful has the app been?
   1. I haven’t used it
   2. No help at all
   3. Slightly helpful
   4. Fairly helpful
   5. Very helpful
   6. Not applicable
6. Overall what do you think about the Milk Man app? (open text)
